# Present and future Köppen-Geiger climate classification maps at 1-km resolution

**DOI:** 10.1038/sdata.2018.214

**Published:** 2018-10-30

**Authors:** Hylke E. Beck, Niklaus E. Zimmermann, Tim R. McVicar, Noemi Vergopolan, Alexis Berg, Eric F. Wood

**Affiliations:** 1 Princeton University, Department of Civil and Environmental Engineering, Princeton, NJ, USA; 2 Swiss Federal Research Institute WSL, CH-8903 Birmensdorf, Switzerland; 3 Department of Environmental Systems Science, Swiss Federal Institute of Technology ETH, Zürich, Switzerland; 4 CSIRO Land and Water, Canberra, ACT, Australia; 5 Australian Research Council Centre of Excellence for Climate System Science, Sydney, Australia

**Keywords:** Ecology, Climate sciences

## Abstract

We present new global maps of the Köppen-Geiger climate classification at an unprecedented 1-km resolution for the present-day (1980–2016) and for projected future conditions (2071–2100) under climate change. The present-day map is derived from an ensemble of four high-resolution, topographically-corrected climatic maps. The future map is derived from an ensemble of 32 climate model projections (scenario RCP8.5), by superimposing the projected climate change anomaly on the baseline high-resolution climatic maps. For both time periods we calculate confidence levels from the ensemble spread, providing valuable indications of the reliability of the classifications. The new maps exhibit a higher classification accuracy and substantially more detail than previous maps, particularly in regions with sharp spatial or elevation gradients. We anticipate the new maps will be useful for numerous applications, including species and vegetation distribution modeling. The new maps including the associated confidence maps are freely available via www.gloh2o.org/koppen.

## Background & Summary

The Köppen-Geiger system classifies climate into five main classes and 30 sub-types. The classification is based on threshold values and seasonality of monthly air temperature and precipitation. Considering vegetation as “crystallized, visible climate”^1^, this classification aims to empirically map biome distributions around the world: different regions in a similar class share common vegetation characteristics. The first version of this classification was developed in the late 19th century^2^; it is still widely used today, for many applications and studies conditioned on differences in climatic regimes, such as ecological modeling or climate change impact assessments^3–8^. This wide use reflects the fact that climate has since long been recognized as the major driver of global vegetation distribution^9–11^. In species distribution models^12^, climate variables are considered the primary driver to explain species ranges at larger spatial extents, while habitat and topography are considered to only be modifiers of plant species distributions at smaller extents^13–15^. The Köppen-Geiger climate classification is a highly suitable means to aggregate complex climate gradients into a simple but ecologically meaningful classification scheme. It is therefore often used as input when analyzing the distribution^4,16,17^ or growth behavior^18^ of species, or to set-up dynamic global vegetation models^19^.

Three recent versions of the world maps of the Köppen-Geiger climate classification exist^20–22^. Kottek *et al.*^20^ produced a map (0.5° resolution) based on CRU TS 2.1^23^ for temperature and VASClimO V1.1^24^ for precipitation. CRU was based on approximately 7000–17,000 stations (depending on the year) and VASClimO on 9343 stations. The Peel *et al.* map^21^ (0.1° resolution) was derived from 4844 air temperature stations and 12,396 precipitation stations. Kriticos *et al.*^22^ produced a map (0.083° resolution) based on WorldClim V1 temperature and precipitation datasets^25^, which are based on 24,542 and 47,554 stations, respectively.

All maps have a relatively low resolution (≥0.1°) and the map of Peel *et al.*^21^ has not been explicitly corrected for topographic effects, which influences air temperature^26^ and precipitation^27^ in mountainous regions. In addition, the maps of Kottek *et al.*^20^ and Peel *et al.*^21^ are based on a relatively small number of stations. This can lead to widespread misclassifications, particularly in regions with a low station density and/or strong climatic gradients such as mountain ranges^28^. Moreover, since these maps do not include corresponding uncertainty estimates, they may provide users a false sense of confidence.

**Figure 1:**
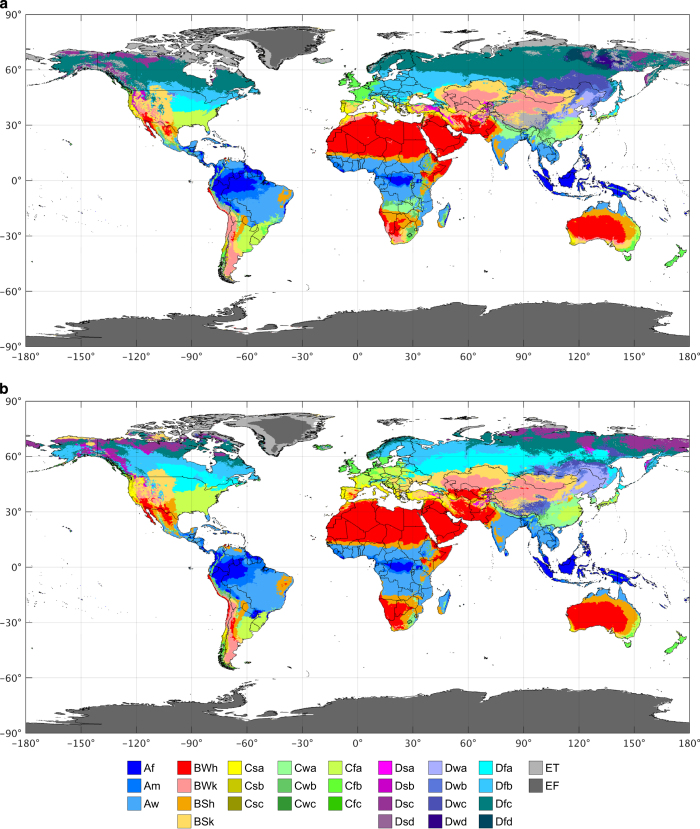
New and improved Köppen-Geiger classifications. Part (**a**) shows the present-day map (1980–2016) and panel (**b**) the future map (2071–2100). The color scheme was adopted from Peel *et al.*^21^.

Here, we present a new and improved Köppen-Geiger climate classification map for the present (1980–2016) with an unprecedented 0.0083° resolution (approximately 1 km at the equator), providing more accurate representation of highly heterogeneous regions (Fig. 1a). To maximize the accuracy and assess uncertainties in map classifications, we combine climatic air temperature and precipitation data from multiple independent sources, including WorldClim V1 and V2, CHELSA V1.2, and CHPclim V1 (Table 1). These datasets have all been explicitly corrected for topographic effects and, with the exception of the CHELSA V1.2 temperature dataset, been based on a large number of stations (≥34,542 for precipitation and ≥20,268 for temperature). The use of multiple data sources allows us to provide an estimate of uncertainty in the derived classes. Further, we combine climate change projections from 32 Coupled Model Intercomparison Project phase 5 (CMIP5^29^) models to map future (2071–2100) climate classes at the same spatial resolution (Fig. 1b).

## Methods

### Köppen-Geiger climate classification

We follow the Köppen-Geiger climate classification as described in Peel *et al.*^21^, which was also used by Kriticos *et al.*^22^ (Table 2). This classification is identical to that presented by Köppen in 1936^1^ with three differences. First, temperate (C) and cold (D) climates are distinguished using a 0 °C threshold instead of a 3 °C threshold, following the suggestion of Russell^30^. Second, the arid (B) sub-climates W (desert) and S (steppe) were identified depending on whether 70% of precipitation occurred in summer or winter. Third, the sub-climates s (dry summer) and w (dry winter) within the C and D climates were made mutually exclusive by assigning s when more precipitation falls in winter than in summer and assigning w otherwise. Note that the tropical (A), temperate (C), cold (D), and polar (E) climates are mutually exclusive but may intersect with the arid (B) class. To account for this, climate type B was given precedence over the other classes.

### Climate data

The present Köppen-Geiger classification map was derived from three climatic datasets for air temperature (WorldClim V1 and V2, and CHELSA V1.2) and four climatic datasets for precipitation (WorldClim V1 and V2, CHELSA V1.2, and CHPclim V1; Table 1). All datasets have a 0.0083° resolution with the exception of CHPclim V1.2, which has a 0.05° resolution. For consistency CHPclim V1.2 was downscaled to 0.0083° using bilinear interpolation.

The future Köppen-Geiger classification was produced using monthly historical and future air temperature and precipitation data from the CMIP5 archive^29^. For the future scenario, we used Representative Concentration Pathway 8.5 (RCP8.5^31^). All climate models with data during the 1980–2016 and 2071–2100 periods were used. Data for 1980–2016 was derived by concatenating historical runs (which end in 2005) and future runs (which begin in 2006). For each model, we only considered a single initialization ensemble. In total 32 models had sufficient data and hence were used for deriving the future map (Table 1).

### Present-day Köppen-Geiger map

The present-day Köppen-Geiger map (Fig. 1a) was derived from an ensemble of high-resolution climatic datasets (Table 1) using the criteria listed in Table 2. Since the climatic datasets have inconsistent temporal coverages, we first adjusted them to reflect the period 1980–2016. To this end, we calculated, for each climatic dataset, monthly 0.5° climatologies for temperature using CRU TS V4.01 and for precipitation using GPCC FDR V7, for both the 1980–2016 period and the temporal span of the climatic dataset. Next, for each month we calculated climate change offsets (for temperature) or factors (for precipitation) between the two periods, and resampled these offsets or factors from 0.5° to 0.0083° resolution using bilinear interpolation, and adjusted the climatic maps by addition (for temperature) or multiplication (for precipitation).

**Figure 2:**
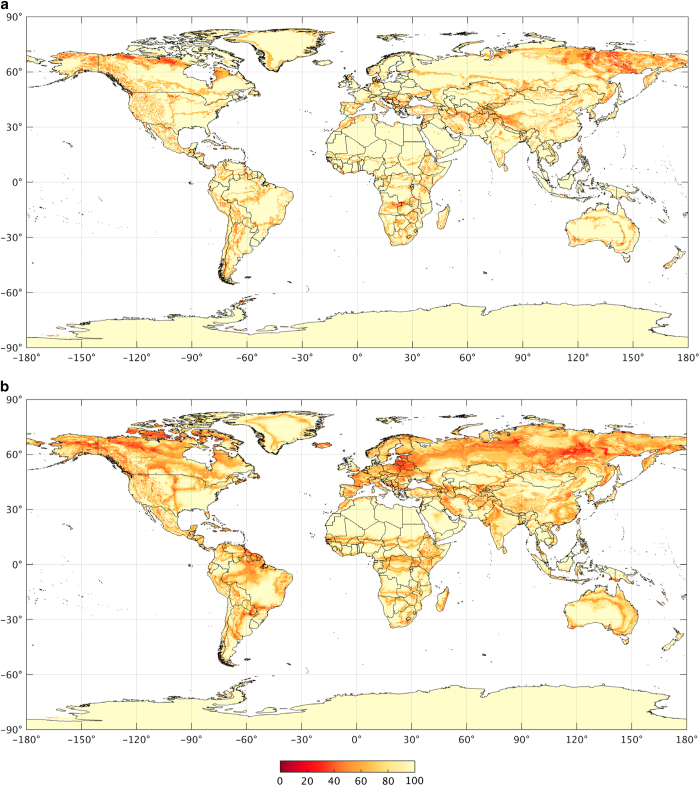
The confidence levels (%) associated with the new Köppen-Geiger classifications. Part (**a**) shows the present-day confidence map (1980–2016) and panel (**b**) the future confidence map (2071–2100). These maps provide an indication of classification accuracy.

For each adjusted temperature and precipitation climatic dataset combination, we derived a Köppen-Geiger map at 0.0083° resolution. From this ensemble of 4×3=12 maps we derived a final map by selecting, for each grid-cell, the most common class (Fig. 1a). A corresponding confidence map was derived by dividing the frequency of occurrence of the most common class by the ensemble size and converting these fractions to percentages (Fig. 2a). For example, if Csa is the most common class for a particular grid-cell, and it has been assigned eight times out of 12, the resulting confidence level is $100\times \frac{8}{12}=66.6$%. This confidence level should be interpreted as the degree of trust we place in our final present-day classification. Confidence levels are generally lower in the vicinity of borders between climate zones, in particular at high latitudes where the climatic data show more uncertainty.

### Future Köppen-Geiger map

**Figure 3:**
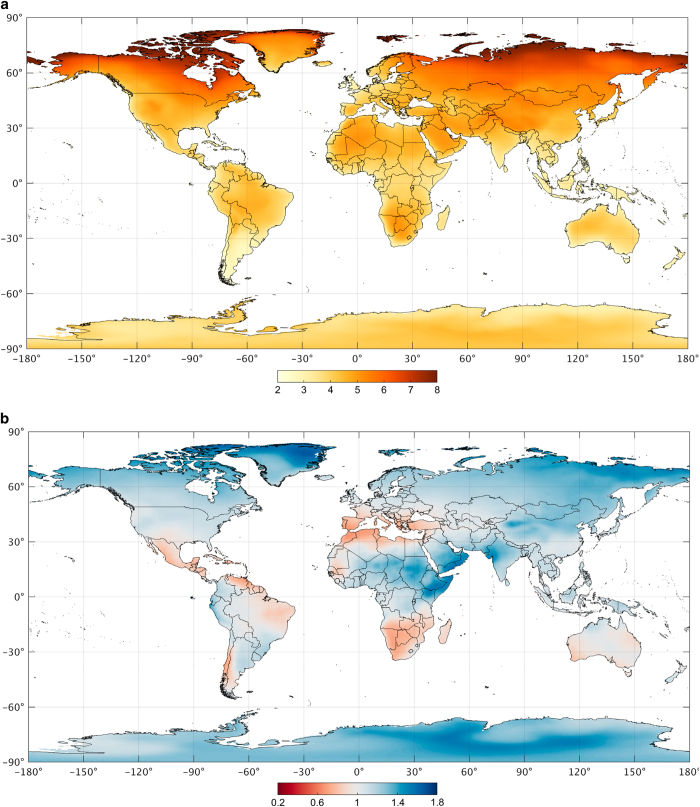
Projected changes in mean air temperature (°C) and precipitation (unitless) between 1980–2016 and 2071–2100 derived from climate model outputs. Part (**a**) presents air temperature change offsets and part (**b**) precipitation change factors. The values represent the mean over all models and months.

The future Köppen-Geiger map (Fig. 1b) was derived by the so-called “anomaly method”^32^ based on an ensemble of climate projections from the 32 CMIP5 models (Table 1). First, observed monthly present-day reference temperature and precipitation climatologies (0.0083° resolution) were derived, by simple averaging of the ensemble of temporally-homogenized, high-resolution climatic maps. Then, for each climate model and each month, we subsequently calculated climate change offsets (for temperature) or factors (for precipitation) between 1980–2016 and 2071–2100 and resampled these offsets or factors from the native model resolution to 0.0083° using bilinear interpolation (Fig. 3). Finally, future high-resolution climatic temperature and precipitation maps were derived from the present-day, observed reference climatologies by addition of the offsets (for temperature) or multiplication by the factors (for precipitation). We want to emphasize that the change factors are never excessively high (i.e., >5; Fig. 3), because (i) model simulations tend to overestimate the precipitation frequency^33^ (resulting in the near-absence of areas with close to zero precipitation), and (ii) over the majority of arid regions the future projections tend toward drying rather than wetting^34^ (resulting in factors <1).

For each climate model, we derived a future Köppen-Geiger map at 0.0083° resolution from the downscaled future temperature and precipitation data. From this ensemble of 32 maps we derived a final map by selecting, for each grid-cell, the most common class (Fig. 1b). A corresponding confidence map was derived by dividing the frequency of occurrence of the most common class by the ensemble size and converting these fractions to percentages (Fig. 2b). For example, if Cfa is the most common class for particular grid-cell, and it has been assigned 24 times out of 32, the corresponding confidence level is $100\times \frac{24}{32}=75.0$%. This confidence level should be interpreted as the degree of trust we have in our final future classification based on the uncertainties in climate change projections. Thus, uncertainties are larger than for the present-day map. In particular, they are larger at high latitudes because of the greater model spread in projected warming in those regions.

### Code availability

The new Köppen-Geiger classifications have been produced using MathWorks MATLAB version R2017a. The function used to classify the temperature and precipitation data according to the criteria listed in Table 2 (KoppenGeiger.m) is freely available via (Data Citation 1) and www.gloh2o.org/koppen. The other codes are available upon request from the first author.

## Data Records

The present and future Köppen-Geiger classification maps and the corresponding confidence maps are freely available for download at (Data Citation 1) and www.gloh2o.org/koppen. The maps are stored in GeoTIFF format as unsigned 8-bit integers. We also provide a legend file (legend.txt) linking the numeric values in the maps to the Köppen-Geiger climate symbols and providing the color scheme used for displaying the maps in the current study (adapted from Peel *et al.*^21^). The maps are referenced to the World Geodetic Reference System 1984 (WGS 84) ellipsoid and made available at three resolutions (0.0083°, 0.083°, and 0.5°; approximately 1 km, 10 km, and 50 km at the equator, respectively). The classifications are upscaled from 0.0083° to 0.083° and 0.5° using majority resampling and the confidence levels using bilinear averaging. Table 3 presents the file naming convention. The maps can be visualized and analyzed using most Geographic Information Systems (GIS) software (e.g., QGIS, ArcGIS, and GRASS).

## Technical Validation

We validated the new high-resolution present-day Köppen-Geiger classification (Fig. 1a), and previous maps from Kottek *et al.*^20^, Peel *et al.*^21^, and Kriticos *et al.*^22^, by calculating the classification accuracy (defined as the percentage of correct classes) using station observations as reference. An initial database was compiled from the Global Historical Climatology Network-Daily (GHCN-D) database^35^ and the Global Summary Of the Day (GSOD) database (https://data.noaa.gov). For each station, we calculated monthly mean temperature and precipitation time series (discarding months with <25 daily values), and subsequently monthly climatologies by averaging the monthly means (if ≥10 values were present). Stations with gaps in the climatologies or missing data for one of the four maps were discarded, resulting in a final dataset comprising 22,078 stations which we used to calculate the classification accuracy of each map.

The newly derived high-resolution present-day Köppen-Geiger classification (Fig. 1a) exhibited a classification accuracy of 80.0%, while the maps of Kottek *et al.*^20^, Peel *et al.*^21^, and Kriticos *et al.*^22^ exhibited classification accuracies of 66.1, 70.9, and 73.4%, respectively. These results confirm that the new map is more accurate, which is primarily due to its high (1 km) resolution and use of an ensemble of topographically-corrected climatic datasets. The map of Kottek *et al.*^20^ showed the lowest classification accuracy, due to its low (0.5°) resolution. The map of Peel *et al.*^21^ also performed less well, due to a lack of topographic corrections and the use of a relatively small number of stations.

We also tested the usefulness of the confidence map associated with the new present-day classification (Fig. 2a) using station observations. We obtained a mean confidence level of 92.6% for the correctly classified stations (*n*=17,667) and 77.4% for the misclassified stations (*n*=4411). The mean confidence level was thus substantially lower for the misclassified stations, confirming that the confidence map provides a useful indication of the classification accuracy.

**Figure 4:**
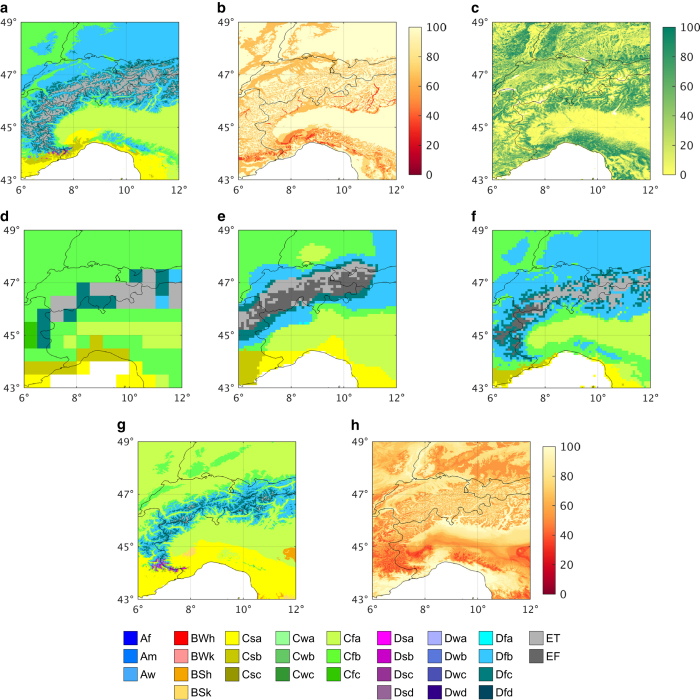
Köppen-Geiger classifications, and associated maps, for the European Alps. Part (**a**) present-day results from our study (1980–2016); (**b**) confidence levels associated with our present-day map; and (**c**) forest cover map^36^ (2000). Historic Köppen-Geiger classification maps for the three previous studies are provided as: (**d**) Kottek *et al.*^20^ (1951–2000); (**e**) Peel *et al.*^21^ (1916–1992); and (**f**) Kriticos *et al.*^22^ (1960–1990). Our future Köppen-Geiger map (2071–2100) is presented in (**g**) and the corresponding confidence map in (**h**). The representative period of each map is listed in parentheses. Thin black lines are country borders and the unmapped white area is part of the Mediterranean Sea.

**Figure 5:**
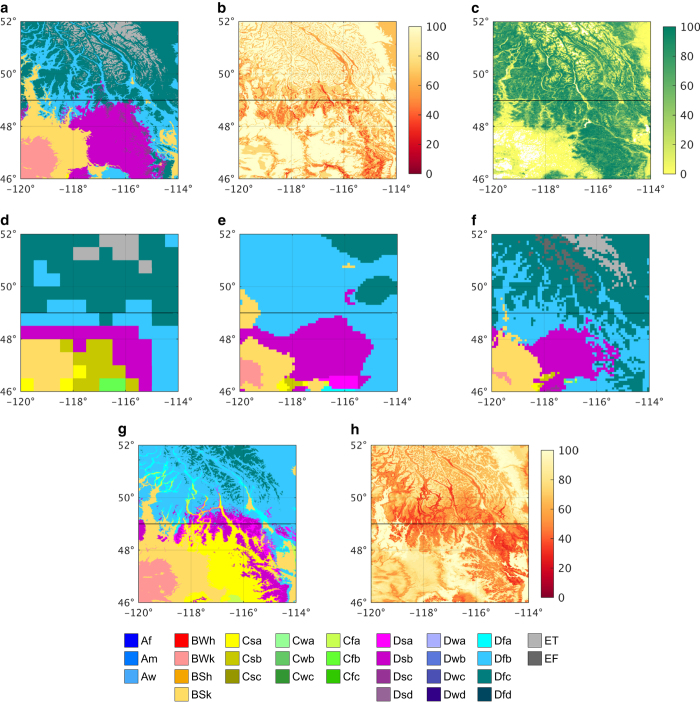
Köppen-Geiger classifications, and associated maps, for the central Rocky Mountains (North America). Part (**a**) present-day results from our study (1980–2016); (**b**) confidence levels associated with our present-day map; and (**c**) forest cover map^36^ (2000). Historic Köppen-Geiger classification maps for the three previous studies are provided as: (**d**) Kottek *et al.*^20^ (1951–2000); (**e**) Peel *et al.*^21^ (1916–1992); and (**f**) Kriticos *et al.*^22^ (1960–1990). Our future Köppen-Geiger map (2071–2100) is presented in (**g**) and the corresponding confidence map in (**h**). The representative period of each map is listed in parentheses. The thick black line at 49° latitude represents the Canada-US border.

Figures 4 and 5 show historic Köppen-Geiger classification maps from all three previous studies and our present-day map for the Alps (Europe) and the central Rocky Mountains (North America), respectively, illustrating the enhanced detail in our map. The other maps sometimes fail to depict important topographic features; the map of Peel *et al.*^21^, for example, does not represent the Apennine mountains (Italy), due to a lack of topographic corrections (Fig. 4e). The new map (Figs. 4a and 5a) also exhibits better agreement with a Landsat-based forest cover map^36^ (30-m resolution; Figs. 4c and 5c). The spatial extent of the polar (E) climate, for example, corresponds closely with treelines in the forest cover maps. Additionally, the new present-day and future Köppen-Geiger maps (Fig. 4a and g, respectively) agree well with equivalent high-resolution maps derived for the Alps^8^ (their Figs. 1 and 2, respectively).

## Usage Notes

The future Köppen-Geiger classification (Fig. 1b) should be viewed as providing insights into potential spatial changes in regional climatic zones under climate change. However, caution should be exerted not to equate those changes directly with changes in actual biomes. First, vegetation changes by 2100 may lag the change in climate zones. Secondly, factors not accounted for in the Köppen-Geiger classification, such as higher atmospheric CO_2_ levels, may alter the relationship between climate classes and vegetation. It is thus advised to interpret the future Köppen-Geiger classification first and foremost from a ‘climatic conditions’ perspective.

The rationale for using the anomaly method to build future maps using climate model projections, instead of directly computing present and future maps from model outputs, is that superimposing future modeled anomalies onto the observed climate removes mean biases from climate model outputs. This is a widely used method in climate change impact assessments^32^. However, an unavoidable limitation of this approach is that because of model spatial biases, modeled climate change anomalies may not be fully geographically consistent with the baseline observed climatology to which they are added^37^ (e.g., if the climate of one region in a given model is spatially shifted relative to reality).

Another irredeemable limitation is that because of their coarser resolution (typically 1–2°), climate model outputs do not resolve future climate change at the same scale as our baseline climatology. Thus, in cases where there could be significant heterogeneities in precipitation change and/or warming below the model resolution (e.g., along coastlines and/or in regions with strong land-cover differences and/or elevation gradients), future changes at the 1-km scale might be under- or over-estimated, because only the model-scale mean anomalies are used to compute future changes. High-elevation mountainous regions are a prime example of this because they are expected to experience considerably more warming than adjacent valleys^38^.

## Additional information

**How to cite this article**: Beck, H. E. *et al.,* Present and future Köppen-Geiger climate classification maps at 1-km resolution. *Sci. Data*. 5:180214 doi: 10.1038/sdata.2018.214 (2018).

**Publisher’s note**: Springer Nature remains neutral with regard to jurisdictional claims in published maps and institutional affiliations.

## Supplementary Material



## Figures and Tables

**Table 1:** Global monthly datasets used for deriving the Köppen-Geiger maps.

**Short name**	**Full name and details**	**Variable(s)**	**Temporal span**	**Spatial resolution**	**Reference(s)**
*High-resolution climatic datasets*					
CHELSA V1.2	Climatologies at High resolution for the Earth’s land Surface Areas (CHELSA) V1.2 (http://chelsa-climate.org)	*T*, *P*	1979–2013	0.0083°	^28^
CHPclim V1	Climate Hazards Group’s Precipitation Climatology (CHPclim) V1 (http://chg.geog.ucsb.edu/data/CHPclim/)	*P*	1980–2009	0.05°	^39^
WorldClim V1	WorldClim V1 (http://www.worldclim.org)	*T*, *P*	1960–1990	0.0083°	^25^
WorldClim V2	WorldClim V2 (http://www.worldclim.org)	*T*, *P*	1970–2000	0.0083°	^40^
*Time-varying datasets used to adjust the climatic data to reflect the 1980-2016 period*					
CRU TS V4.01	Climatic Research Unit (CRU) TimeSeries (TS) V4.01 (https://crudata.uea.ac.uk/cru/data/hrg/)	*T*	1901–2016	0.5°	^41^
GPCC FDR V7	Global Precipitation Climatology Centre (GPCC) Full Data Reanalysis (FDR) V7 extended using First Guess (https://www.dwd.de/EN/ourservices/gpcc/gpcc.html)	*P*	1951–present	0.5°	^42,43^
*Time-varying dataset used to derive the future map*					
CMIP5	Coupled Model Intercomparison Project Phase 5 (CMIP5) historical and future (RCP8.5) data for 32 climate models^a^ (https://esgf-node.llnl.gov/projects/esgf-llnl/)	*T*, *P*	1850–2100	Varies	^29^
Variable definitions: *T* = air temperature; *P* = precipitation. ^a^The following climate models were used (initialization ensemble between parentheses): ACCESS1-0 (r1i1p1), ACCESS1-3 (r1i1p1), bcc-csm1-1 (r1i1p1), bcc-csm1-1-m (r1i1p1), BNU-ESM (r1i1p1), CCSM4 (r1i1p1), CESM1-BGC (r1i1p1), CESM1-CAM5 (r1i1p1), CESM1-CAM5-1-FV2 (r1i1p1), CMCC-CESM (r1i1p1), CMCC-CM (r1i1p1), CMCC-CMS (r1i1p1), CSIRO-Mk3-6-0 (r7i1p1), FGOALS-g2 (r1i1p1), FGOALS-s2 (r3i1p1), FIO-ESM (r1i1p1), GISS-E2-H-CC (r1i1p1), GISS-E2-R (r1i1p1), GISS-E2-R-CC (r1i1p1), inmcm4 (r1i1p1), IPSL-CM5A-LR (r1i1p1), IPSL-CM5A-MR (r1i1p1), IPSL-CM5B-LR (r1i1p1), MIROC-ESM (r1i1p1), MIROC-ESM-CHEM (r1i1p1), MIROC5 (r1i1p1), MPI-ESM-LR (r1i1p1), MPI-ESM-MR (r1i1p1), MRI-CGCM3 (r1i1p1), MRI-ESM1 (r1i1p1), NorESM1-M (r1i1p1), and NorESM1-ME (r1i1p1).					

**Table 2:** Overview of the Köppen-Geiger climate classes including the defining criteria.

**1st**	**2nd**	**3rd**	**Description**	**Criterion**^a^
A			Tropical	Not (B) & *T_cold_*≥18
	f		- Rainforest	*P_dry_*≥60
	m		- Monsoon	Not (Af) & *P_dry_*≥100-*MAP*/25
	w		- Savannah	Not (Af) & *P_dry_*<100-*MAP*/25
B			Arid	*MAP*<10×*P_threshold_*
	W		- Desert	*MAP*<5×*P_threshold_*
	S		- Steppe	*MAP*≥5×*P_threshold_*
		h	- Hot	*MAT≥*18
		k	- Cold	*MAT<*18
C			Temperate	Not (B) & *T_hot_*>10 & 0<*T_cold_*<18
	s		- Dry summer	*P_sdry_<40* & *P_sdry_<P_wwet_/3*
	w		- Dry winter	*P_wdry_<P_swet_/10*
	f		- Without dry season	Not (Cs) or (Cw)
		a	- Hot summer	*T_hot_*≥22
		b	- Warm summer	Not (a) & *T_mon__10_*≥4
		c	- Cold summer	Not (a or b) & 1≤*T_mon__10_*<4
D			Cold	Not (B) & *T_hot_*>10 & *T_cold_≤*0
	s		- Dry summer	*P_sdry_<*40 & *P_sdry_<P_wwet_*/3
	w		- Dry winter	*P_wdry_<P_swet_*/10
	f		- Without dry season	Not (Ds) or (Dw)
		a	- Hot summer	*T_hot_*≥22
		b	- Warm summer	Not (a) & *T_mon__10_*≥4
		c	- Cold summer	Not (a, b, or d)
		d	- Very cold winter	Not (a or b) & *T_cold_*<-38
E			Polar	Not (B) & *T_hot_*≤10
	T		- Tundra	*T_hot_*>0
	F		- Frost	*T_hot_*≤0
Adapted from Peel *et al.*^21^. ^a^Variable definitions: *MAT* = mean annual air temperature (°C); *T_cold_* = the air temperature of the coldest month (°C); *T_hot_* = the air temperature of the warmest month (°C); *T_mon__10_* = the number of months with air temperature >10 °C (unitless); *MAP* = mean annual precipitation (mm y^−1^); *P_dry_* = precipitation in the driest month (mm month^−1^); *P_sdry_* = precipitation in the driest month in summer (mm month^−1^); *P_wdry_* = precipitation in the driest month in winter (mm month^−1^); *P_swet_* = precipitation in the wettest month in summer (mm month^−1^); *P_wwet_* = precipitation in the wettest month in winter (mm month^−1^); *P_threshold_*=2×*MAT* if >70% of precipitation falls in winter, *P_threshold_=*2×*MAT*+28 if >70% of precipitation falls in summer, otherwise *P_threshold_*=2×*MAT*+14. Summer (winter) is the six-month period that is warmer (colder) between April-September and October-March.				

**Table 3:** File naming convention.

**Filename**	**Spatial resolution**	**Dimensions (rows × columns)**	**Description**
0p0083.tifBeck_KG_V1_present_0p083.tif 0p5.tif	0.0083°0.083°0.5°	21600 × 432002160 × 4320360 × 720	Present-day (1980–2016) Köppen-Geiger climate classification
0p0083.tifBeck_KG_V1_present_conf_0p083.tif 0p5.tif	0.0083°0.083°0.5°	21600 × 432002160 × 4320360 × 720	Confidence level in the present (1980–2016) Köppen-Geiger classification expressed as percentage
0p0083.tifBeck_KG_V1_future_0p083.tif 0p5.tif	0.0083°0.083°0.5°	21600 × 432002160 × 4320360 × 720	Future (2071–2100) Köppen-Geiger climate classification
0p0083.tifBeck_KG_V1_future_conf_0p083.tif 0p5.tif	0.0083°0.083°0.5°	21600 × 432002160 × 4320360 × 720	Confidence level in the future (2071–2100) Köppen-Geiger classification expressed as percentage
