# Publisher Correction: Present and future Köppen-Geiger climate classification maps at 1-km resolution

**DOI:** 10.1038/s41597-020-00616-w

**Published:** 2020-08-17

**Authors:** Hylke E. Beck, Niklaus E. Zimmermann, Tim R. McVicar, Noemi Vergopolan, Alexis Berg, Eric F. Wood

**Affiliations:** 1 Princeton University, Department of Civil and Environmental Engineering, Princeton, NJ USA; 2 Swiss Federal Research Institute WSL, CH-8903 Birmensdorf, Switzerland; 3 Department of Environmental Systems Science, Swiss Federal Institute of Technology ETH, Zürich, Switzerland; 4 CSIRO Land and Water, Canberra, ACT Australia; 5 Australian Research Council Centre of Excellence for Climate System Science, Sydney, Australia

Correction to: *Scientific Data* 10.1038/sdata.2018.214, published online 30 October 2018

Due to a processing error during production, colours in the PDF version of Fig. 1 were not accurately rendered. The HTML version remains accurate. A copy of Fig. 1 is included below, and is accurate in both the HTML and PDF versions of this correction notice.

**Fig. 1:**
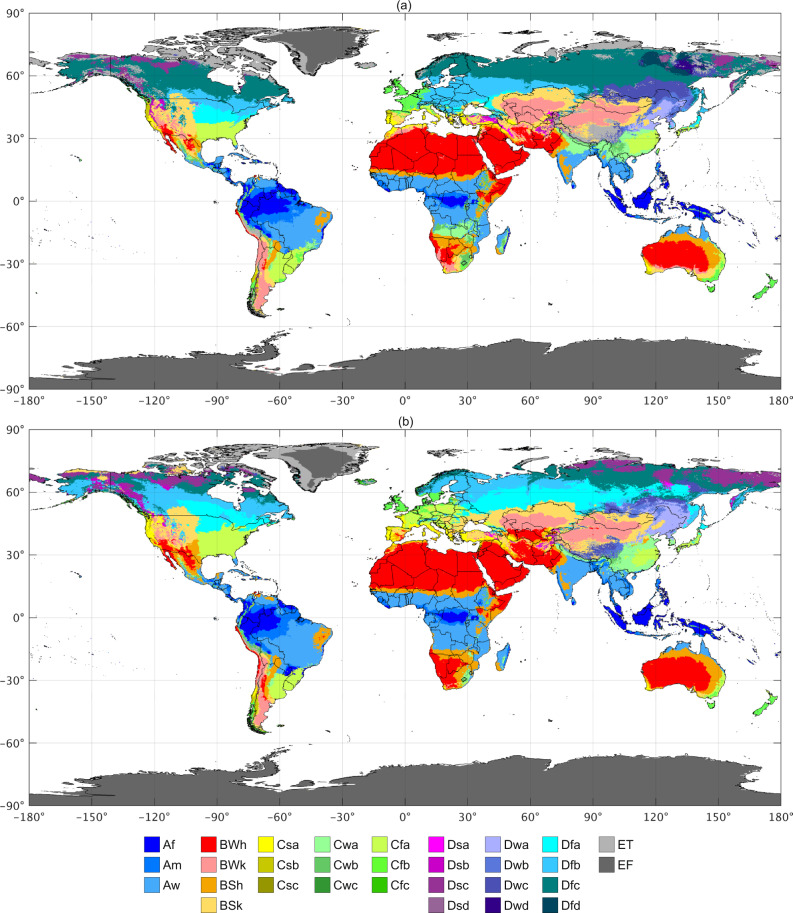
New and improved Köppen-Geiger classifications.

